# Relative Energy Deficiency in Sport (REDs) and Body Composition

**DOI:** 10.3390/sports14080314

**Published:** 2026-07-23

**Authors:** Mark Godley, Daniel Fink, Tim Saupe, Edgars Edelmers, Liāna Pļaviņa, Māra Pilmane

**Affiliations:** Department of Morphology, Rīga Stradiņš University, LV-1010 Riga, Latvia; 060269@rsu.edu.lv (M.G.); 061269@rsu.edu.lv (D.F.); 060816@rsu.edu.lv (T.S.); liana.plavina@rsu.lv (L.P.); mara.pilmane@rsu.lv (M.P.)

**Keywords:** low energy availability, relative energy deficiency, body composition, athletes, bone mineral density, muscle mass, visceral fat, hormonal adaptations, nutritional strategies, hydration status

## Abstract

Background: Relative energy deficiency in sport (REDs) is a multi-system syndrome precipitated by sustained low energy availability (LEA) and known to perturb bone, muscle, adipose, endocrine, and fluid–electrolyte homeostasis in athletic populations. Research question: How does chronic exercise-induced LEA modify the four principal compartments of body composition—bone mineral density, skeletal muscle mass, adipose (total and visceral) tissue, and hydration status—and to what extent do these modifications differ across endurance, strength, and aesthetic sporting disciplines? Objective: The objective of this narrative review was to synthesise the post-2000 peer-reviewed literature addressing this question, to delineate sex- and sport-specific patterns of compositional change, and to appraise the evidence base underpinning current nutritional and screening recommendations. Methods: A structured search of PubMed, Web of Science, Scopus, and Google Scholar (January 2000–June 2026) was performed; 85 sources specific to REDs, body composition, and athletes met the pre-specified inclusion criteria and informed the synthesis (104 references are cited in total, including background and methodological sources), the reporting of which was guided by the SANRA framework. Scope: The review integrates physiological, endocrine, and nutritional evidence across both sexes, contrasts REDs risk across endurance, ultra-endurance, weight-category/aesthetic, speed–power, and team disciplines and addresses practical implications for screening, interdisciplinary care, and future research.

## 1. Introduction

Energy availability is a central determinant of physiological function and training adaptation in athletes. Formally, it is defined as the dietary energy remaining to support non-exercise physiological processes after exercise energy expenditure has been subtracted, expressed per kilogram of fat-free mass per day. When this residual energy falls below approximately 30 kcal·kg FFM^−1^·day^−1^, endocrine, skeletal, and metabolic homeostases are disturbed. In athletic populations, this mismatch between energy intake and the combined demands of training and basal physiology is now formally recognised as relative energy deficiency in sport (REDs) [1,2]. Throughout this review, the term athlete denotes competitive athletes from national to international levels, recreationally trained individuals following a structured training programme, and physically active adults aged 16 years or older; where a finding applies to only one of these groups, the population is named explicitly at the point of citation.

Recent epidemiological work indicates that LEA and clinical REDs affect a substantial minority of athletes; contemporary prevalence estimates in endurance and aesthetic disciplines commonly range from 20% to over 60%, with the highest risk reported in sports emphasising leanness or weight categories [3,4]. The consequences of REDs extend beyond competitive performance. The population most consistently reported as susceptible in the primary literature comprises adult athletes aged approximately 18–40 years, a range that encompasses the conventional window of peak athletic performance and within which the clinical sequelae of exercise-induced LEA on body composition and intermediary metabolism have been most frequently characterised [5,6]. Documented sequelae include neuroendocrine dysregulation, reductions in bone mineral density, chronic fatigue, and an elevated incidence of musculoskeletal injury [7,8]. Because several of these manifestations are subclinical in their initial presentation, early recognition is frequently delayed. Converging evidence further indicates that even moderate, cumulative energy deficits can elicit measurable physiological perturbations. The magnitude and reversibility of these perturbations appear to be duration-dependent: short-term exposure (≤1 month) and prolonged exposure (>6 months) produce mechanistically distinct effects, as summarised by Stellingwerff and Hamilton [9]. Given the sustained growth in participation in endurance, aesthetic, and weight-category sports, athletes increasingly operate at the physiological ceiling of training load. A quantitative characterisation of how such loading, when coupled with inadequate energy intake, alters the composition and function of skeletal muscle, adipose, and bone tissue is therefore essential for evidence-based prevention and for optimising training adaptation. Updated international consensus work and recent syntheses [2,3,10] now call for standardised screening, tiered severity staging, and sex-specific prevention strategies. The present narrative review is aligned with that direction.

### 1.1. Overview of Existing Research

Many studies have examined how body composition changes under energy deficiency. The most affected areas include bone density, muscle mass, body and visceral fat, and hydration levels.

The short summaries that follow are included purely as an organising preview of the four physiological domains treated in depth in Section 3, Section 4, Section 5 and Section 6; they are not intended as a standalone literature review. Each domain is developed with full citations, mechanistic discussion, and updated contemporary evidence in the corresponding substantive section below.

- Bone Density: Low energy intake also weakens bones. Without sufficient energy, osteoblast (bone-forming) activity slows while osteoclast (bone-resorbing) activity increases. This leads to reduced bone mineral density (BMD), increasing the risk of stress fractures and, over time, osteoporosis. This is common in sports like running, ballet, and cycling [11]. Although the female athlete triad initially focused on women [12], it is now understood that male athletes can also develop poor bone health when under-fuelled [13]. DXA-derived reference values for BMD in the healthy general population aged 18–40 years are approximately 1.10–1.16 g/cm^2^ in females and 1.18–1.19 g/cm^2^ in males [14].
- Muscle Mass: Muscle is another area that suffers. When energy is low, the body may start breaking down muscle to create energy. This leads to muscle loss, slower recovery, and reduced strength. Also, levels of key hormones that help build muscle, like IGF-1 and testosterone, are often lower during energy deficiency [7,15]. Athletes in strength sports or those requiring endurance are especially affected. Muscle mass peaks between 20 and 30 years old and then starts to decline after the age of 30–40 years old. Skeletal muscle typically accounts for approximately 40–50% of total body mass in healthy young adults, although this proportion varies with sex, discipline, and the method of assessment [7,15].
- Body Fat: During energy deficiency, adipose tissue is among the first compartments to decline, as the body mobilises fat stores when dietary intake is insufficient. Although a lower fat mass may benefit endurance or appearance in certain sports, excessive loss—particularly below healthy levels—can suppress essential hormones such as leptin, oestrogen, and testosterone. These changes can impact reproductive health, bone strength, and immune function. Reference ranges reported for non-athletic adults aged 18–40 years are approximately 8–19% body fat in men and 18–28% in women [8,16].
- Visceral Fat: Visceral fat (around the organs) is also reduced. This may sound beneficial, but losing too much can disrupt hormonal balance and metabolism. Research by Loucks [17] shows that very low visceral fat can cause issues with the thyroid and reproductive systems.
- Hydration Status: Athletes with low energy availability are often also inadequately hydrated, whether through deliberate fluid restriction to control weight or through reduced thirst secondary to low energy intake. Poor hydration adds physiological strain, impairs concentration, and raises the risk of heat illness, particularly in endurance sports [18]. Total body water is typically 50–60% of body mass in healthy adults [18,19]. More recent experimental work using contemporary body-water methodology confirms the 50–60% range in healthy adults while highlighting that within-training fluid shifts of ≥2% body mass meaningfully impair both neuromuscular and endurance performance [20,21].

These figures are general-population reference ranges. They are derived predominantly from dual-energy X-ray absorptiometry (bone mineral density and body fat) and from dilution or bioimpedance methods (total body water) in non-athletic adults, and they are quoted here only to orient the reader to the compartments discussed below. They are not athlete-specific and should not be read as targets or as diagnostic thresholds: sex, age, discipline, training status, and the assessment method itself shift the expected values materially, and DXA, bioimpedance, and skinfold-based estimates are not interchangeable [14,16,22,23]. Deviation from these ranges is not, in itself, evidence of REDs.

Most of these studies examine one component at a time, but few consider the overall framework of body composition under exercise-induced energy deficiency. The present review integrates these components within a single framework and contrasts their behaviour across endurance, strength, and aesthetic disciplines.

### 1.2. Publication Search Strategy

The present work is a narrative literature review. A structured search was conducted between September 2024 and February 2025, and updated in June 2026, in four electronic databases—PubMed, Scopus, Web of Science (Core Collection) and Google Scholar—supplemented by hand-searching of the reference lists of the most relevant retrieved articles. A narrative rather than systematic design was chosen deliberately. The purpose of this review is integrative: to bring four compartments of body composition into a single conceptual frame and to grade the strength of the evidence within each. The underlying studies are too heterogeneous in design, population, and outcome metrics to support quantitative pooling, and a systematic review restricted to a single comparable outcome would have excluded precisely the cross-compartment comparison the review exists to make. The search is therefore reported in structured form for transparency and reproducibility, not as a claim to systematic status. Because the review is narrative rather than systematic, no formal PRISMA compliance is claimed; the reporting of the narrative itself was guided by the six SANRA criteria [24], which informed the description of the search, the referencing, and the presentation of evidence. We do not report a self-assessed SANRA score: scoring by the authors is at material risk of bias, and a favourable self-score would not, in any case, substitute for the reader’s own appraisal. The limitations that constrain the strength of our conclusions—the absence of quantitative pooling, methodological heterogeneity between the included studies, and the scarcity of direct evidence in the adipose and hydration domains—are stated explicitly in Section 2.4, Section 2.5 and Section 6 rather than absorbed into a summary score.

Searches were guided by three concept blocks: (A) low energy availability, LEA, relative energy deficiency in sport, RED-S, REDs, and female athlete triad; (B) body composition, bone mineral density, muscle mass, muscle protein synthesis, visceral fat, hydration, and total body water; and (C) athlete*, sport*, exercise, and training. Records were limited to peer-reviewed material published between 1 January 2000 and 30 June 2026, available in English full text.

Eligibility was defined a priori. Articles were considered for inclusion if they were peer-reviewed original research, narrative or systematic reviews, or international consensus statements reporting on competitive or recreationally trained athletes, or physically active adults aged ≥ 16 years, and addressed at least one outcome relevant to energy availability, energy balance, body composition, bone, muscle, endocrine, or fluid–electrolyte status. Editorials, conference abstracts, grey literature, popular-science sources, animal-only studies, records confined to non-athletic clinical populations without an exercise component, and records for which the full text could not be retrieved were excluded.

Records retrieved across the four databases were exported to a shared reference manager, duplicates were removed, and titles and abstracts were screened against the eligibility criteria; records passing title/abstract screening were retrieved in full and re-assessed against the same criteria. Screening was performed independently by three of the authors, and disagreements were resolved by discussion until consensus was reached. Because the REDs framework has evolved rapidly since the 2014 IOC consensus, with major updates in 2018 and 2023, post-2020 peer-reviewed literature was prioritised as the anchor evidence for clinical interpretation, while foundational mechanistic and threshold-setting work [11,25,26] was retained for continuity with the primary literature. Approximately one-third of the finally included sources were published between 2021 and 2026. No formal meta-analysis was undertaken, consistent with the narrative nature of the review.

The following key words and combinations of terms were used in the search process:

- “Low energy availability”
- “Relative energy deficiency in sport”
- “REDs”
- “Body composition”
- “Bone mineral density”
- “Muscle protein synthesis”
- “Energy deficiency and athletes”
- “Exercise energy availability”
- “Fat-free mass”
- “Hydration status”
- “Visceral fat”
- “Sport-specific prevalence”

A total of 85 sources met the full-text inclusion criteria specific to REDs, body composition, and athletes and were retained for the primary analysis; 22 of these were identified in the June 2026 search update, which extended the coverage of hydration, visceral adiposity, fat-free mass, and sport-specific prevalence. An additional 19 background and methodological references were cited in the manuscript but were not derived from the primary database search, bringing the total reference count to 104. A summary of the screening process is provided in Table 1.

To orient the reader to the evidence base underpinning the following discussion, Table 2 summarises a representative subset of the included studies, grouped by design (e.g., expert consensus, narrative reviews, and original experimental/observational work).

## 2. How Low Energy Availability Affects Body Fat, Visceral Fat, Muscle Mass, Bone Mass, and Hydration

### 2.1. LEA and the Manifestations of REDs

Two conceptually distinct constructs underpin the quantitative framework for energy status in athletes: energy availability (EA) and energy balance (EB). Although frequently discussed together, these constructs must not be treated as interchangeable. EA is a physiological construct that isolates the dietary energy remaining to support non-exercise physiological processes after subtraction of exercise energy expenditure, normalised to fat-free mass (kcal·kg FFM^−1^·day^−1^). EB is a whole-body energetic construct that expresses the net difference between total energy intake and total energy expenditure, and it is not normalised to body composition. Consequently, an athlete may achieve a neutral EB while simultaneously sustaining a pathologically LEA whenever exercise energy expenditure is high, which is precisely why EA—and not EB—is the relevant index for identifying and staging REDs.

Energy availability (EA) is defined as the amount of dietary energy left for physiological processes after subtracting the energy used during exercise training. It is calculated using the following formula:

$$EA=\frac{\mathrm{Energy}\;\mathrm{Intake}\;(\mathrm{EI})-\mathrm{Exercise}\;\mathrm{Energy}\;\mathrm{Expenditure}\;(\mathrm{EEE})}{\mathrm{Fat}\;\mathrm{Free}\;\mathrm{Mass}\;(\mathrm{FFM})}$$

It is expressed in kilocalories per kilogram of FFM per day [2].

Energy availability refers to the amount of energy needed to maintain cellular homeostasis, regulate body temperature, facilitate growth and reproductive processes, and support immune function [17]. Energy balance, by contrast, is the difference between total energy intake (EI) and total energy expenditure (TEE) [17] (see equation below).

Fat-free mass (FFM) is a measure of all anatomical components within the human body that exclude fat, including muscle, bone, organs, and water [16]. It can be estimated using several methods; however, dual-energy X-ray absorptiometry (DXA) is widely regarded as the reference standard for assessing bone mineral density and soft tissue composition due to its high precision and low measurement error [16]. Other approaches, such as bioelectrical impedance analysis (BIA), air-displacement plethysmography (ADP), and skinfold-based models, are more accessible and practical in field and clinical settings, but they generally show lower accuracy and larger prediction errors when compared with DXA [22,23].

EB=EI−TEE

**Figure 1:**
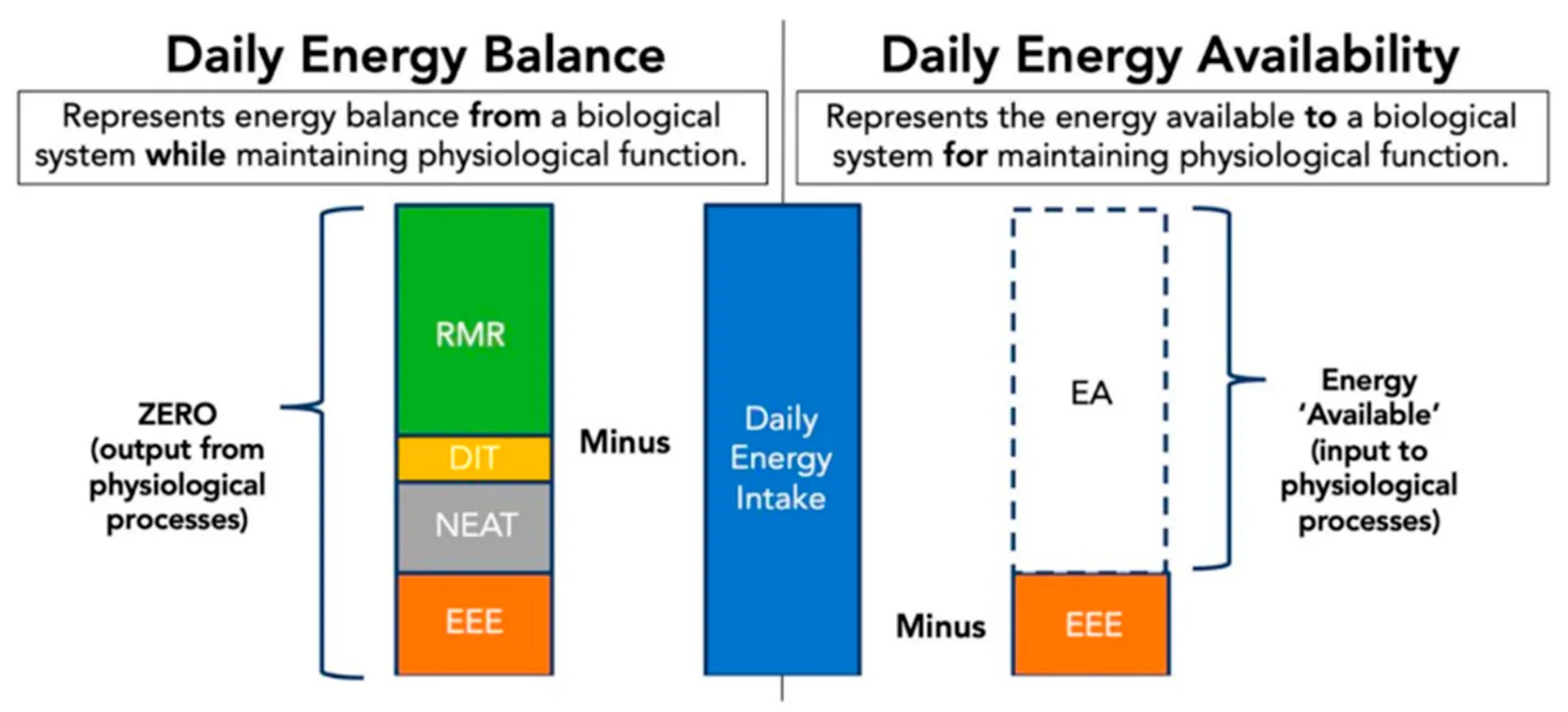
Daily energy balance and daily energy availability. Figure created by the authors; the concept is based on ref. [33].

Here, TEE is a cumulative total of the resting metabolic rate, the thermic effect of food, exercise energy expenditure, and non-exercise activity thermogenesis [32]. A positive energy balance (EB) generally results in weight gain, while a negative EB leads to weight loss (Figure 1).

To guide clinical and performance-related decision-making, research has aimed to establish an optimal threshold value for energy availability of approximately 45 kcal per kilogram of FFM per day to support healthy physiological functions [32]. By contrast, studies also indicate that an EA below 30 kcal/kg FFM per day, even for short periods, can disrupt several vital systems.

The findings of Loucks and Thuma’s [26] trial showed that regularly menstruating women who experienced short-term EA below 30 kcal/kg FFM per day exhibited signs of altered thyroid hormone levels, suppressed luteinising hormone (LH), and increased markers of bone resorption. These findings also highlighted that low energy availability (LEA) can acutely impair endocrine and skeletal systems, even when there are no apparent energy deficits or changes in body mass.

It is important to note that most trials have been primarily based on female athletes, as male athletes may have different sensitivities to LEA; nevertheless, the physiological effects are increasingly recognised in both sexes [7,27]. The development and severity of LEA are influenced by other factors such as:

- Type and volume of exercise;
- Duration of LEA exposure;
- Body composition and fat-free mass;
- Macronutrient distribution;
- Biological sex;
- Individual variability [27].

LEA can be either intentional (such as in sports that emphasise leanness or weight categories) or unintentional—resulting from poor nutrition planning or inadequate recovery during intense training periods. It can occur with or without a diagnosable eating disorder and therefore requires targeted screening [7]. Historically, concerns about LEA were addressed through the female athlete triad, which included: low energy availability (with or without disordered eating), menstrual dysfunction, and low bone mineral density [34]. Although the triad was an essential step into the development and understanding of LEA, it focuses solely on female athletes. It does not account for the full range of physiological effects now associated with LEA. In response, the International Olympic Committee (IOC) introduced the broader and more inclusive framework of relative energy deficiency in sport (REDs). REDs recognises that LEA can affect athletes of all genders and disciplines and acknowledges its impact on multiple physiological systems [2] and has since been complemented by a dedicated physiological model [35]. REDs is associated with impairment across multiple physiological systems:

- Endocrine (e.g., HPA axis, HPG axis, and thyroid hormones)
- Skeletal (e.g., increased bone resorption and reduced BMD)
- Cardiovascular (e.g., bradycardia and hypotension)
- Immunological (e.g., increased susceptibility to illness)
- Gastrointestinal (e.g., slowed motility and bloating)
- Haematological (e.g., iron-deficiency anaemia)
- Neuromuscular (e.g., reduced strength and coordination)
- Psychological (e.g., mood disturbances, anxiety, and depression)

**Figure 2:**
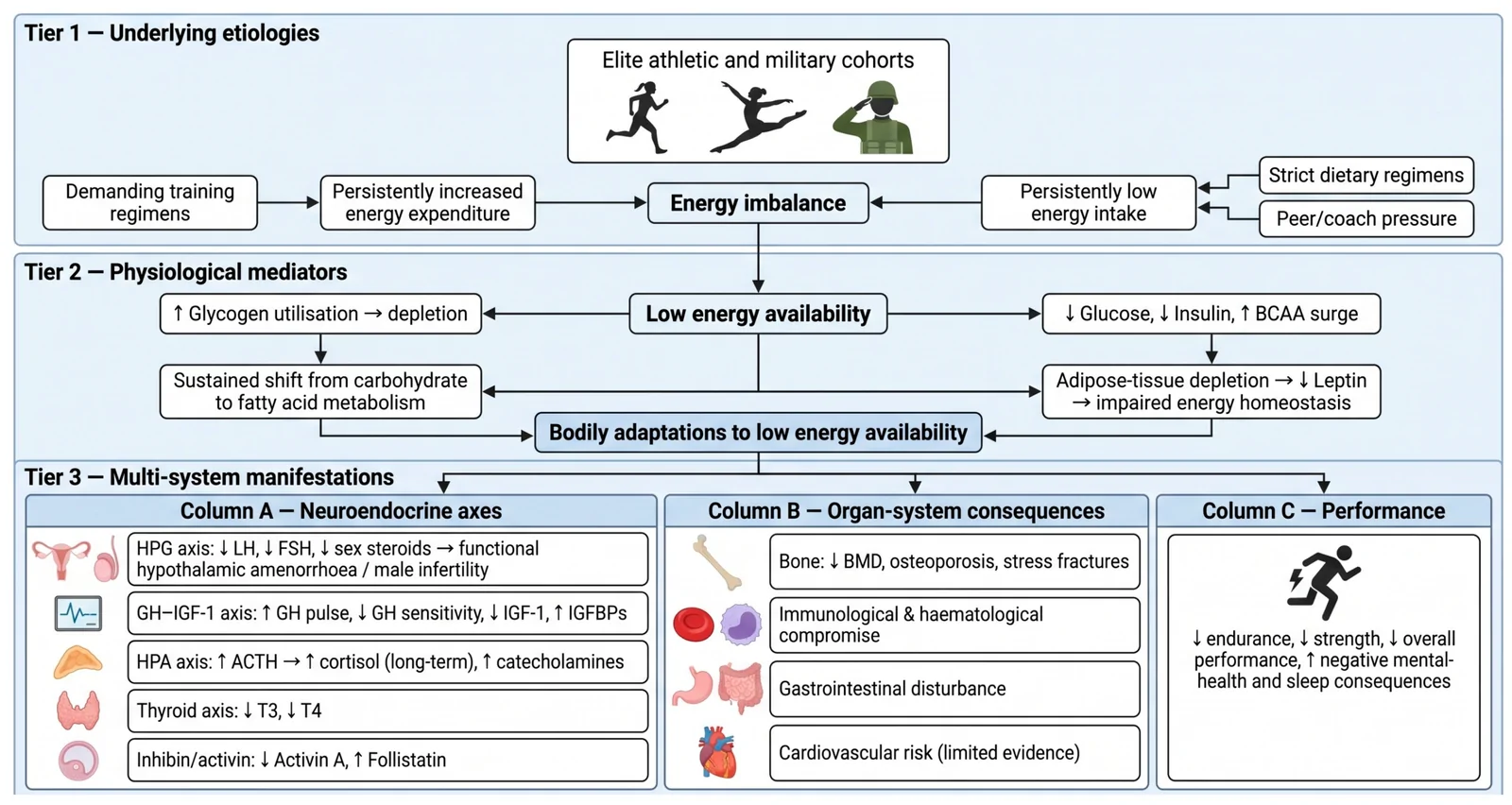
Long-term and short-term energy availability and its impact on several physiological systems. Arrows (→) denote a causal or sequential relationship between elements; upward (↑) and downward (↓) arrows denote an increase or decrease in the indicated parameter, respectively. Figure created by the authors; the concept is based on ref. [10].

These changes can have a negative impact not only on health but also on sports performance, including diminished training adaptation, poor recovery, and higher risk of injury [2,10] (Figure 2).

Given the complexity and variability in responses to LEA, there is no universal threshold that guarantees protection from REDs. Nonetheless, maintaining EA at or above 45 kcal/kg FFM/day is widely recommended, and EA below 30 kcal/kg FFM/day is consistently associated with adverse outcomes. Therefore, understanding and monitoring EA should be a top priority for athlete health, performance enhancement, and prevention of long-term physiological damage [2].

### 2.2. How Relative Energy Deficiency in Sport (REDs) and Low Energy Availability (LEA) Affect Bone Density

Bone health is an essential part of athletic performance and long-term well-being. In athletes, bones are influenced not only by training and exercise but also by the amount of energy available to the body after accounting for exercise demands. Physical load, and therefore intensity level, is quantified using the metabolic equivalent of task (MET), where physical activities are assigned MET values that represent a multiple of resting energy expenditure. Walking at a moderate pace may be roughly 3–4 METs, whereas more intense activity such as running can exceed 8 METs [36]. When athletes do not eat enough to match the energy they spend, this creates a state called LEA. Over time, LEA can affect many body systems, including hormones, metabolism, and especially bone. The broader condition of REDs describes the health and performance problems associated with LEA. Both concepts are strongly linked to reduced bone mineral density (BMD), higher risk of stress fractures, and weaker bone structure.

To assess the impact of LEA and REDs on bone density, EB, exercise effects, hormonal changes, dysfunction models (e.g., female athlete triad and REDs), gender differences, and sport-specific risks must all be considered.

Energy balance refers to the relationship between dietary energy intake (DEI) and energy expenditure. When intake matches expenditure, body weight remains stable. If intake is lower than expenditure, the body enters a negative energy balance, which over time can reduce body mass [17,37]. Adequate EA is needed for normal body functions, including bone growth and repair [37].

Exercise typically builds and strengthens bone, particularly sports that involve impact and varied loading (for example, sports such as gymnastics, basketball, and weightlifting increase bone mineral density), due to the stress placed on bone tissue. Sports with multidirectional impacts—such as gymnastics—stimulate new bone growth the most [38].

By contrast, endurance sports like long-distance running, swimming, and cycling are less effective for bone health. While running provides some impact, the high training volumes combined with LEA risk can reduce bone strength. For instance, a high proportion of adolescent elite Kenyan female runners (aged approximately 13–20 years, mean ~16 years) have been reported to be at risk of energy deficiency, with associated menstrual dysfunction and disordered-eating behaviours [39], and reduced bone mineral density has been documented in elite Kenyan runners [40]. Swimming and cycling are non-weight-bearing, which means they place little stress on the skeleton, and therefore are less osteogenic. Male jockeys also show reduced bone mass and higher bone resorption markers due to restricted food intake to maintain racing weight [41]. Because these observations were derived largely from cross-sectional comparisons, they should be interpreted as associations rather than proof of causation; collectively, however, they suggest that chronic LEA may attenuate the osteogenic benefits of exercise in both sexes.

When energy intake does not meet the demands of training, bones are negatively affected. LEA lowers levels of bone growth hormones such as insulin, leptin, triiodothyronine (T3), and insulin-like growth factor 1 (IGF-1) [25,26]. This disrupts the balance between bone growth and bone breakdown.

Research using bone markers shows that LEA increases bone resorption (breakdown) while reducing bone formation. For example, Ihle and Loucks [42] showed that when women reduced their EA to very low levels (30, 20, and 10 kcal·kg LBM^−1^·day^−1^), bone formation decreased and bone breakdown increased.

Two main models explain how LEA affects bone health:

- **Female Athlete Triad**: Originally defined as disordered eating, amenorrhea, and osteoporosis, it now refers to a spectrum ranging from normal to impaired energy availability, menstrual function, and bone health [11,43].
- **REDs:** Recognises that low energy availability affects not only bone and reproductive function but also cardiovascular, metabolic, and psychological health [2].

Both models demonstrate how insufficient nutrition for exercise might result in significant long-term skeletal issues.

Women are generally more affected by LEA, partly due to the impact on reproductive hormones like oestrogen, which is essential for bone protection. However, studies suggest men are not entirely immune. While some research shows men may be more resistant to short-term LEA, likely because of protective androgens and the absence of menstrual energy costs, long-term LEA still damages their bones [13]. Findings are mixed: some research shows altered bone markers under LEA, while others show no immediate effect. Variability may be higher in men, but over time, chronic energy deficiency leads to bone loss in both sexes.

Low energy availability and REDs pose serious threats to athletes’ bone health. While exercise typically builds bone, when paired with insufficient fuelling, it can damage skeletal integrity. Short-term LEA disrupts bone metabolism, while long-term LEA reduces bone density, damages microarchitecture, and increases fracture risk [39,42]. Women appear more vulnerable due to reproductive hormone changes, but men are also at risk, particularly in sports requiring strict weight control.

Understanding and avoiding LEA are critical to athlete health. Coaches, players, and medical personnel should keep a careful eye on energy availability and ensure that physical demands are met with sufficient nutrition to safeguard bone health and overall performance.

### 2.3. How Relative Energy Deficiency in Sport (REDs) and Low Energy Availability (LEA) Affects Muscle Mass

Reducing body mass, according to many athletes and coaches, can enhance exercise performance and the power-to-body mass ratio. On the other hand, short-term severe LEA causes energy-conserving responses that result in decreased insulin-like growth factor I hormone levels, decreased resting metabolic rate, impaired protein turnover in collagen-rich tissues, and suppression of the hypothalamic–pituitary–ovarian axis in women.

**Figure 3:**
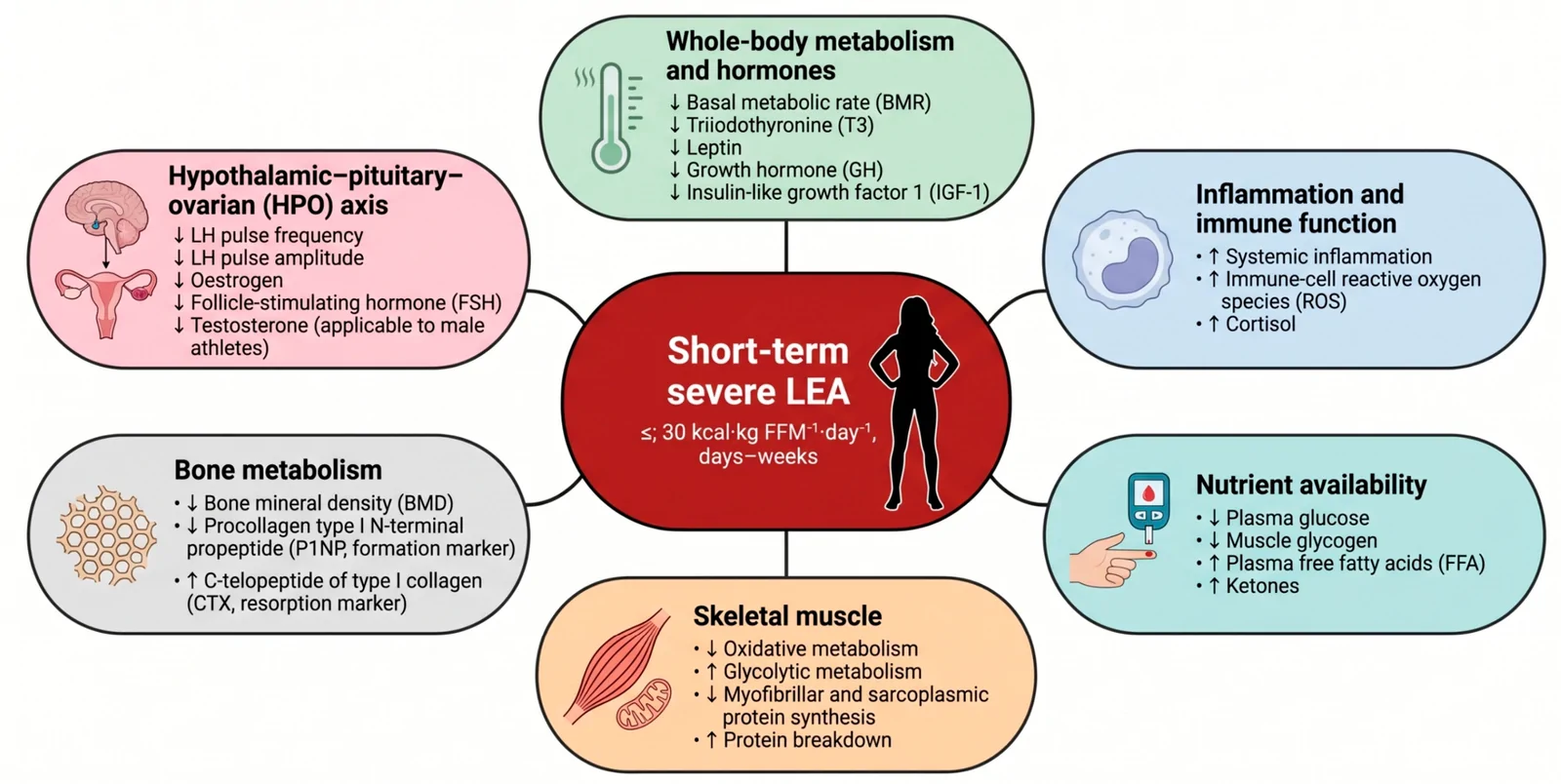
Short-term severe LEA. Upward (↑) and downward (↓) arrows denote an increase or decrease in the indicated parameter relative to adequate energy availability, respectively. Figure created by the authors; the concept is based on ref. [30].

The negative effects of short-term severe LEA in female athletes have also been highlighted by recent studies, which have shown decreased muscle protein synthesis, elevated cortisol levels, enhanced fat utilisation during exercise, and direct impairments in power, sprinting, and endurance exercise performances despite body mass reductions [30] (Figure 3).

Skeletal muscle represents a highly plastic, metabolically active tissue vital for athletic performance. According to studies on energy deficiency, the resting mixed muscle fractional synthetic rate (FSR) of physically active adults, measured by isotope infusion, decreased after 10 days on a 20% energy deficit [44]. A more recent study also confirmed this finding, showing that daily integrated myofibrillar protein synthesis rate, measured by deuterium oxide (D2O) ingestion, was lower in adult males experiencing 10 days on a 40% energy deficit compared with a 5-day energy balance period [45].

The studies mentioned above suggest that reductions in muscle protein synthesis occur when EA is decreased. However, a direct comparison between energy balance/deficiency and EA cannot be made [32] since EA varies considerably between individuals when energy intake is limited to the same amount in calories or as a percentage of total energy requirement.

The impact of LEA on muscle protein turnover has only been examined in one study. According to Areta et al. [46], young resistance-trained males and females who were fed a diet equivalent to an EA of 30 kcal·kg FFM^−1^·day^−1^ for 5 days had a 27% decrease in resting myofibrillar muscle protein synthesis, as measured by isotope infusion, as opposed to 45 kcal·kg FFM^−1^·day^−1^. Moreover, the study showed that a single bout of resistance exercise restored myofibrillar protein synthesis levels and that adding one bolus of protein supplementation increased myofibrillar protein synthesis above rates observed at rest in EA of 45 kcal·kg FFM^−1^·day^−1^. These results indicate that LEA might decrease acute muscle protein synthesis when measured in the overnight fasted state, but resistance exercise and protein supplementation may lessen this effect.

Because a state of LEA in athletes usually develops over weeks or even months rather than being short-term [2], Oxfeldt et al. [28] proposed investigating the effects of 10 days of LEA on daily integrated myofibrillar and sarcoplasmic muscle protein synthesis in young, trained females engaging in supervised exercise training (including both resistance and cardiovascular exercise). This was compared with optimal energy availability (OEA) and matched dietary protein intake (2.2 g·kg lean mass^−1^·day^−1^). They used D2O to measure protein synthesis over time under free-living conditions. They quantified both myofibrillar and sarcoplasmic muscle protein FSR to gain insights into proteins directly associated with the contractile apparatus and sarcoplasmic proteins involved in enzymatic functions, mitochondrial activity, and other intracellular processes. Oxfeldt et al. [28] calculated changes in body composition using various methods, such as X-ray absorptiometry, resting metabolic rate, muscle biopsies, blood samples, urinary nitrogen balance, saliva samples, and checking myofibrillar and sarcoplasmic muscle protein synthesis. The results indicated that both myofibrillar and sarcoplasmic protein synthesis decreased in LEA by 0.08% and 0.2% per day, respectively, and that fat- and bone-free lean mass decreased by 0.4 kg over 10 days of LEA despite resistance exercise and high dietary protein availability to stimulate anabolic processes.

LEA is likely connected to the reported downregulation of key anabolic hormones such as insulin, T3, or IGF-I [47], which play a crucial role in stimulating muscle protein synthesis [48]. Furthermore, the elevated cortisol levels linked to LEA may worsen the effects of reduced insulin, IGF-I, and T3 levels by further inhibiting anabolic signalling pathways like mTOR and promoting protein degradation, thereby fortifying the catabolic environment in skeletal muscle [49].

Fat-free mass (FFM) warrants separate consideration because it is simultaneously the tissue most directly eroded by sustained under-fuelling and the denominator against which energy availability is itself expressed. LEA is defined as the state in which insufficient dietary energy remains to support physiological function once the energy cost of exercise has been met [50]; expressing that residual per kilogram of FFM presupposes that the FFM compartment is stable, which under prolonged energy deficiency it is not. FFM is the single largest determinant of the resting metabolic rate, and therefore of daily energy requirement, and it is positively associated with energy intake and with self-reported hunger across lean, overweight, and obese populations—an association that appears to be mediated through RMR rather than through fat mass [51,52].

The capacity of adipose tissue to release energy is finite: theoretical and empirical work indicates a ceiling on the rate at which energy can be transferred from the fat store during hypophagia [53]. Once a dietary deficit exceeds that ceiling—as it may in lean athletes with little fat mass to mobilise—the shortfall is met increasingly by the oxidation of lean tissue, producing a measurable reduction in FFM. Because FFM is the principal contributor to RMR, its loss lowers resting energy expenditure further, and the calculated EA of an athlete may consequently appear to stabilise even as the underlying tissue base contracts [53,54]. Reported FFM in athletes should therefore be tracked longitudinally rather than treated as a fixed normalising constant, since a falling FFM both signals and perpetuates energy deficiency. This reasoning is consistent with the measured 0.4 kg loss of fat- and bone-free lean mass observed by Oxfeldt et al. [28] over only 10 days of LEA despite resistance training and generous protein provision.

### 2.4. Does Relative Energy Deficiency in Sport (REDs) and Low Energy Availability (LEA) Affect Body and Visceral Fat?

Although fat loss and energy restriction improve markers of lipid metabolism, inflammation, and insulin sensitivity in the general population [55], in athletes they raise serious concerns.

**Figure 4:**
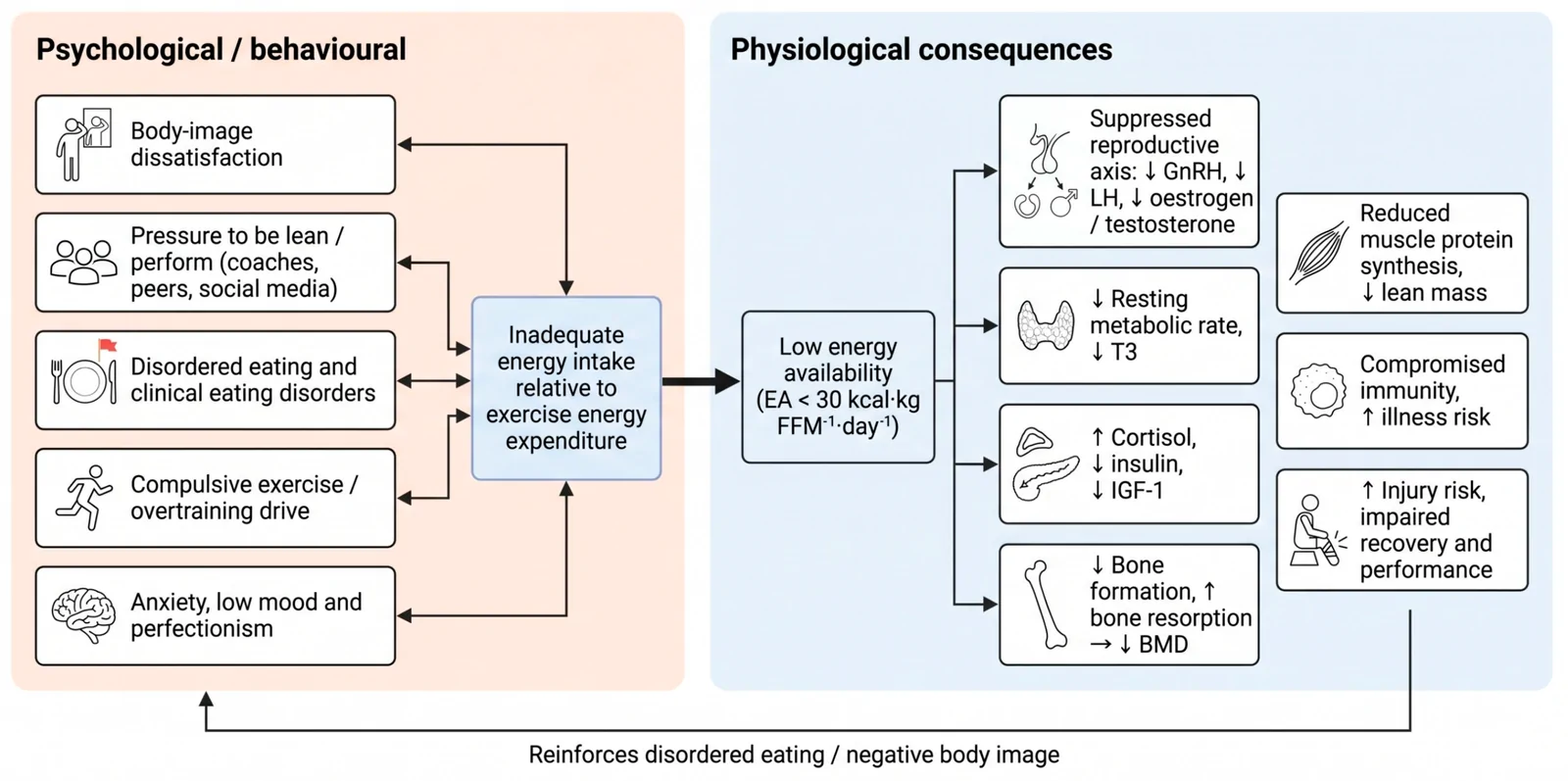
Physiological and psychological mechanisms in LEA. Arrows (→) denote a causal or directional relationship; upward (↑) and downward (↓) arrows denote an increase or decrease in the indicated parameter, respectively. The bottom arrow illustrates a feedback loop whereby physiological consequences reinforce disordered eating and negative body image. Figure created by the authors; the concept is based on ref. [59].

Athletes can induce LEA due to a mismatch between energy intake and exercise energy expenditure, aimed at optimising body mass or composition for competition, avoiding weight gain during injury and illness, or purely because of eating disorders (EDs). This leads to impaired metabolic functions both in the short and long term, causing disturbances in glucose and lipid metabolism [2]. Some of the most common eating disorders include anorexia nervosa, bulimia nervosa, and binge-eating disorder [56]. These disorders originate from a mental condition that causes disrupted attitudes toward eating, body weight, and shape [30,57,58] (Figure 4).

Current research does not directly link LEA and REDs to changes in body and visceral fat. While studies offer insights into fat regulation and metabolic effects in energy-deficient states, these do not establish causality with LEA/REDs. This represents a hypothesis-generating area requiring targeted human trials in athletes to clarify mechanisms and impacts. The material that follows in this section should therefore be read as indirect and mechanistic rather than as direct evidence in athletes: where LEA and REDs are linked to fat regulation, it is through shared endocrine and metabolic pathways (e.g., leptin and insulin signalling) and through nutritional-intervention or animal-model data, none of which establish a causal effect of LEA/REDs on total or visceral adiposity specifically [60,61]. Statements about fat distribution below are extrapolations from this adjacent literature and are flagged as such.

The adjacent literature is nevertheless informative and is summarised here in outline only. Visceral fat is metabolically and endocrinologically distinct from subcutaneous depots and is the compartment most strongly linked to insulin resistance and systemic inflammation [62,63]. In non-athletic cohorts, caloric restriction reliably reduces visceral adiposity irrespective of the macronutrient composition of the restricted diet [64,65], aerobic exercise shows a dose–response relationship with visceral fat loss [66], and rodent data indicate that this depot is under endocrine as well as purely energetic control [67]. None of this evidence was derived from energy-deficient athletes, and no study has yet quantified visceral adipose tissue in athletes stratified by measured energy availability. Any inference that LEA lowers visceral fat, or that very low visceral fat itself contributes to the endocrine disturbances of REDs through loss of leptin signalling [17], is therefore hypothesis-generating and is advanced strictly as such. This remains the clearest single gap in the adipose compartment of the REDs literature.

Disordered-eating and clinical eating-disorder behaviours are more prevalent among athletes than in the general population and are more common in women [56,68,69]; where a drive for leanness combines with high training loads comprise a recognised route into LEA [11,70]. During LEA, the body preferentially supports survival processes such as cellular maintenance and thermoregulation over growth and reproduction [71].

Where body-mass reduction is nonetheless pursued, generalised guidance for leaner athletes recommends a gradual rate of 0.5–1% per week to preserve RMR [72], FFM [73,74], sex hormones, and reproductive function [74,75], interpreted with caution across different sports.

Any deliberate manipulation of EA should therefore be time-limited, medically supervised, and supported by a sports dietitian, with adequate protein (1.6–2.4 g·kg^−1^·day^−1^) and carbohydrate intake maintained throughout.

### 2.5. Does Relative Energy Deficiency in Sport (REDs) and Low Energy Availability (LEA) Affect Hydration?

Having an adequate level of hydration is essential for life. In the human body, water plays a vital role, being distributed both intracellularly (60% of total body water) and extracellularly (40% of total body water). Intracellular water constitutes the cell volume, while extracellular water includes plasma, interstitial fluid, cerebrospinal fluid, and synovial fluid [20]. Water intake is vital for the distribution of body water, as about 50% of total drinking fluids comes from water itself and food. Without water, a human can survive only for a few days [20].

For much of the period covered by this review, no study had examined a direct link between LEA or REDs and hydration status or body-water distribution. As set out below, that position now requires qualification, although hydration remains the least well characterised of the four compartments considered here. Hydration plays a crucial part in body composition and overall health. A study conducted among mice found that muscle is the first organ to lose water, suggesting that fluctuations in hydration status could directly affect muscle function [19]. Since this study, there has been more evidence to indicate the impact of hydration status on neuromuscular function within athletes. According to Cheuvront, Carter, and Sawka [76], dehydration of over 2% of body weight impairs endurance exercise performance, including maximal aerobic capacity, by reducing blood volume and thus blood flow to the muscles [21,77]. However, these effects are independent of LEA/REDs, highlighting another hypothesis-generating area for future athlete-specific research. To be explicit, the mouse model of muscle water loss [19] and the dehydration-performance findings [76] cited above were drawn from contexts unrelated to energy deficiency; they are presented only to illustrate why hydration could plausibly interact with body composition, and they should not be read as evidence that LEA or REDs alter hydration status in athletes.

Emerging evidence suggests that hydration and energy availability may be less independent than previously assumed. Hydration status appears capable of modifying appetite-regulating endocrine signals. Exercising in a dehydrated state has been associated with lower circulating ghrelin concentrations than exercising euhydrated, although in that trial, post-exercise appetite and ad libitum energy intake were not themselves significantly reduced [78]. Any effect of hypohydration on subsequent energy intake is therefore likely to be modest, but its direction is consistent with a mechanism whereby poor fluid status could blunt the drive to eat and thereby deepen an existing energy deficit rather than correct it.

Epidemiological and clinical data point the same way. In a survey of regularly physically active individuals, inadequate hydration emerged as a significant predictor of REDs risk, and the association was most apparent in men [79]—a finding of particular interest given how poorly male-specific risk factors for REDs are currently characterised. Clinical case material illustrates the extreme of the same continuum: in a male mixed martial arts athlete, aggressive weight-making that combined severe energy restriction with deliberate dehydration precipitated relative energy deficiency, hypernatraemia, and acute kidney injury [80], demonstrating that the two exposures are frequently incurred together and that their consequences may be additive rather than merely parallel. Habitual hypohydration before training or competition has likewise been documented in populations already recognised as being at elevated REDs risk, including elite cross-country skiers and dancers, in whom it coexists with insufficient energy and carbohydrate availability [81,82].

Taken together, these observations justify a more explicit position than the earlier literature permitted: hydration is best regarded not as a parallel and unrelated concern but as a potentially modifiable component of the REDs risk profile, particularly in weight-category and aesthetic disciplines in which fluid restriction is used instrumentally to make weight or to alter appearance. The evidence nevertheless remains associative—none of the cited studies manipulated energy availability and hydration status independently—so the direction of causality is undetermined, and controlled trials that dissociate the two exposures are required before hydration can be incorporated into REDs staging with confidence.

A group of athletes (20–35 years old) who take advantage of water hydration levels are bodybuilders, where aesthetics, symmetry, and muscle definition are key evaluation criteria [83]. Balancing intracellular and extracellular water is vital for optimising the physical performance of bodybuilders and the aesthetic look. While extreme water manipulation—for example, forced dehydration—can ruin this balance, resulting in health risks such as reduced muscle strength, fatigue, and increased risk of injury [84], which could theoretically be linked to LEA, this association currently lacks empirical support and warrants targeted investigation in athlete populations.

**Summary**

LEA and REDs create significant concerns in athletes, predominantly among females in middle- and long-distance events, where multiple physiological systems are impaired with and without ED/DE behaviours. The impaired systems create a negative impact on athletes’ health and their sports performance, resulting in poor recovery and a higher risk of injury. The evidence differs sharply in strength across compartments. The links between LEA, bone, and muscle are well supported, whereas any relationship with total and visceral adiposity remains indirect, and the relationship with hydration is emerging and associative; the latter two should be read as hypotheses rather than established contributors to REDs (see Section 2.4, Section 2.5 and Section 6). Bone and muscle mass, however, do have a strong connection to LEA and, if not monitored, can cause detrimental health issues. This is because when energy is in a deficit, the human body will prioritise energy systems such as thermogenesis and cellular maintenance instead of focusing on osteogenesis and muscle growth; therefore, these declines can be a good indicator to suggest medical attention should be sought to ensure the avoidance of long-term health risks of LEA and REDs.

### 2.6. Effects of Low Energy Availability in Endurance, Strength, and Aesthetic Sports

**Endurance Sports:** Endurance athletes (e.g., distance runners and cyclists) are very prone to low energy availability (LEA) due to their high training volumes and caloric expenditures. In this population, LEA often occurs unintentionally when caloric intake does not keep up with exercise demands. The physiological effects of LEA in endurance sports can be very severe. Athletes with chronic LEA experience declines in aerobic performance, such as reduced endurance capacity and slower race times, as well as poor training response. For example, LEA has been linked to decreased running speed, impaired coordination, reduced concentration, and an overall drop in endurance performance in distance athletes. Endurance athletes with LEA also face higher risks of injury and illness. Research indicates that LEA compromises bone health and increases the incidence of bone stress injuries like stress fractures. Immune function may be impaired as well, evidenced by a greater likelihood of illness and upper respiratory infections in athletes with low energy status. Female endurance athletes are especially vulnerable to menstrual dysfunction (amenorrhea) due to LEA, which contributes to low oestrogen and consequent loss of bone density. Male endurance athletes can likewise experience hormonal disruptions. Prolonged energy deficiency in males has been associated with lowered testosterone and metabolic adaptations, although men may require a more severe energy deficit to exhibit reproductive effects. In summary, LEA in endurance sports typically leads to impaired endurance performance and recovery, higher injury risk (particularly bone-related injuries), hormonal disturbances, and immune system suppression [3,13,29].

**Ultra-Endurance Sports:** The energetic demands of ultra-endurance competition magnify each of these mechanisms. Recent synthesis suggests that a substantial proportion of ultra-endurance athletes—by some estimates up to 65%—may be affected by REDs or its precursors, reflecting not only the extreme and often only partially replaceable energy cost of the events themselves but also the sociocultural valorisation of leanness and asceticism within these communities [85]. Sport-specific prevalence data reinforce the ranking: in a cohort of Finnish female national- and international-level athletes, 82% of those competing in endurance disciplines were classified as at risk of REDs and 17.9% as at severe risk, the highest of any sport category examined [86]. Consistent with this, bone quality is measurably poorer in winter endurance athletes presenting with REDs risk factors than in those without [87].

**Aesthetic Sports:** Aesthetic sports include activities such as gymnastics, figure skating, diving, ballet, bodybuilding, and other disciplines where leanness and appearance are emphasised for performance or judging. Due to deliberate dieting or disordered eating to preserve a small figure, athletes participating in aesthetic sports have among of the highest rates of low energy availability and accompanying problems. In contrast to endurance athletes, aesthetic athletes may experience direct external demands to maintain their slenderness (e.g., judges’ ratings, performance apparel, and cultural standards), which can result in a chronic energy deficit.

The effects of LEA on these athletes are profound and multifaceted. As a physical manifestation of the female athlete triad and REDs, female athletes participating in aesthetic sports frequently suffer from amenorrhoea or irregular menstruation due to a lack of energy. This menstrual dysfunction signals disrupted reproductive hormones (e.g., low oestrogen), contributing to decreased bone mineral density and a high incidence of stress fractures and osteoporosis in this group. For example, elite gymnasts and dancers with long-term LEA frequently present with delayed menarche, menstrual cycle disturbances, and reduced bone mass, heightening their risk for bone injuries. In both female and male aesthetic athletes generally between the ages of 20–35 years old [83], LEA is associated with muscle wasting, chronic fatigue, and injuries. These athletes often report persistent fatigue, decreased strength-to-weight ratio over time, and more frequent illnesses or slow healing, all of which impair overall performance quality. Aesthetic sports also have significant psychological consequences. In addition to LEA, eating disorders (ED) may be more common when body image is prioritised. According to studies, athletes who participate in aesthetic sports are much more likely than players in other sports to experience disordered eating and ED. A vicious cycle of deteriorating performance and health results from the combined load of ED and LEA, which exacerbates health problems. Malnutrition also makes energy shortage worse.

Athletes in aesthetic disciplines may not detect short-term performance declines from LEA, since losing weight can initially improve aesthetic line or power-to-weight ratio and thereby disguise the adverse effects. A lighter gymnast, for instance, might achieve higher jumps or a dancer may attain a desired look on stage. However, these perceived benefits are temporary and come at the cost of longer-term consequences. Over time, chronic LEA is commonly associated with stagnating or declining performance as injuries, illness and fatigue accumulate, and any short-term gain is overshadowed by reduced training capacity, mood disturbance and greater injury risk as REDs develops. Thus, in aesthetic sports, the effects of low energy availability manifest in serious health impairments (hormonal, skeletal, and psychological) that ultimately undermine sustained performance [4,88].

**Strength, Speed, and Power Sports:** Athletes in strength, speed, and power disciplines occupy an intermediate position. Their absolute exercise energy expenditure is lower than that of endurance athletes, and LEA in this group is more often episodic—concentrated around weight-making phases, competition preparation, or periods of deliberate restriction—than chronic. The consequences are nevertheless substantial: approximately half of speed and power athletes in a recent national screening reported bone stress injuries, a prevalence only modestly below that recorded in endurance disciplines [86]. Because the primary training stimulus is neuromuscular, the earliest manifestations tend to be suppressed anabolic signalling, reduced myofibrillar protein synthesis, and blunted recovery, rather than the reproductive and skeletal endpoints that dominate the endurance literature [28,46]. These decrements are readily misattributed to overtraining rather than to under-fuelling, which delays recognition.

**Weight-Category Sports:** Disciplines in which competition is organised by body mass—such as combat sports, wrestling, lightweight rowing, and horse racing—superimpose acute weight-making on whatever chronic deficit already exists. The characteristic pattern is rapid weight loss achieved through combined energy and fluid restriction in the days before weigh-in, a practice associated with intentional caloric restriction and disordered eating [89] and, in its extreme form, with relative energy deficiency, hypernatraemia, and acute kidney injury [80]. The episodic nature of the exposure should not be mistaken for benignity since it is typically repeated many times across a season and a career.

**Team Sports:** Team sports have been studied least, and the assumption that intermittent activity is protective against LEA is not well supported. Approximately 36% of elite male volleyball players have been shown to exhibit markers of low energy availability [90], and qualitative work in elite netball identifies high training volumes, limited awareness of REDs among athletes and support staff, and body-image pressure as converging risk factors [91]. Prevalence in team settings therefore appears moderate rather than negligible, and the principal obstacle to recognition is the near-absence of routine screening rather than the absence of risk.

The sport-specific gradient in REDs risk, the mechanisms that generate it, and its most frequent clinical consequences are summarised in Table 3.

**Comparative Perspective:** Although athletes in endurance, strength, and aesthetic sports are negatively impacted by LEA, the precise results and apparent duration of those effects can differ. Strength athletes lose muscle mass and strength and have anabolic hormone suppression, endurance athletes suffer mostly from aerobic performance impairments and bone injuries, and aesthetic athletes have high rates of menstruation and bone density problems in addition to possible eating disorders. Notably, because of the emphasis on leanness, endurance and aesthetic athletes frequently have comparable difficulties. The frequency of LEA and its related health effects are significant in both categories, particularly in females. Strength athletes are somewhat distinct in that LEA is frequently episodic (e.g., pre-competition weight cuts) and primarily degrades neuromuscular performance and recovery. Despite these variations, all three sport categories show that sustained low energy availability raises health risks and impairs athletic performance. This wide range of multisystem impacts in male and female athletes across all activity kinds has been identified as the syndrome known as REDs (relative energy deficiency in sport). In practice, this means coaches and athletes in any discipline should be vigilant about adequate fuelling. Early identification of LEA is crucial: screening tools like the LEAF-Q (Low Energy Availability in Females Questionnaire) or LEAM-Q for males can help flag at-risk athletes before significant performance declines or injuries occur. Ultimately, addressing and preventing LEA through nutrition education, appropriate training loads, and, if needed, medical intervention is essential to maintain both the health and competitive longevity of endurance, strength, and aesthetic sport athletes [2,3].

### 2.7. Underrepresented Populations: Male, Adolescent, and Masters Athletes

The evidence summarised above is drawn overwhelmingly from adult female athletes, and this skew is historical rather than physiological: the female athlete triad framed the field around menstrual function [34], and the reproductive endpoints that follow from that framing are more readily measured in women than in men [27]. Male athletes are therefore under-studied rather than unaffected, and the male data that do exist indicate a different presentation rather than an absence of harm. Four days of severe LEA in exercising men reduced leptin and insulin levels without an acute fall in testosterone or T3 levels [92], suggesting greater short-term resilience of the male reproductive axis. The male athlete triad has nonetheless been formally defined around LEA, suppressed reproductive function, and impaired bone health [13], and chronic energy deficiency in male endurance athletes is associated with lowered testosterone and impaired performance [93]. Two of the findings introduced in this review are male-specific: inadequate hydration predicts REDs risk most clearly in men [79], and the most severe documented consequence of combined energy and fluid restriction—relative energy deficiency with hypernatraemia and acute kidney injury—occurred in a male athlete [80]. The practical corollary is that instruments validated in women cannot simply be transposed: the LEAF-Q has no equally well-validated male counterpart, and the LEAM-Q requires further validation before it can carry the same diagnostic weight.

Adolescent athletes constitute the second major gap. Their risk is elevated because the energy cost of growth and pubertal maturation is superimposed on training load, and because weight manipulation during the years of peak bone accrual can delay puberty and compromise the attainment of peak bone mass [2,43]. The available data are concerning: a high proportion of adolescent elite Kenyan female runners, aged approximately 13–20 years, are at risk of energy deficiency, with associated menstrual dysfunction and disordered eating [39]. Yet adolescents are routinely and properly excluded from the controlled LEA trials that underpin the mechanistic literature, with the consequence that the thresholds and staging criteria applied to them are extrapolated from adults rather than derived from adolescents. This extrapolation is rarely acknowledged when REDs screening is applied in youth sport.

Age is likewise treated as a nuisance variable rather than as an effect modifier. The primary literature concentrates on adults of approximately 18–40 years, the conventional window of peak athletic performance [5,6], with the result that masters athletes—a growing population in endurance disciplines, and one in whom age-related decline in bone mineral density and lean mass would be expected to compound any energy deficit—are almost entirely absent from the REDs evidence base. Until these three populations are studied directly, the REDs framework can reasonably be applied to them, but it should be applied with an explicit acknowledgement that its thresholds were derived elsewhere, and that the confidence established in adult female athletes does not necessarily transfer to other groups.

## 3. Hormonal and Metabolic Adaptations to Low Energy Availability

The endocrine and metabolic consequences of LEA, defined in Section 2.1, are treated here in their own right rather than as a compartment of body composition because they mediate most of the compositional changes described above [2]. For example, the resting metabolic rate (RMR) falls and the reproductive axis slows when energy is scarce [26,31], and the body prioritises survival over growth and reproduction.

### 3.1. Hormonal Adaptations to Low Energy Availability

When energy intake is insufficient for exercise needs, the brain and endocrine system respond quickly. In women, the reproductive axis is very sensitive to LEA: even a few days of severe energy deficit can disrupt the normal pulsatile release of gonadotropin-releasing hormone (GnRH) and luteinising hormone (LH) [26,30]. For example, Loucks and Thuma showed that, in sedentary women, reducing EA below 30 kcal/kg FFM/day caused a big drop in LH pulse frequency and later menstrual cycle changes [26]. Similarly, short-term LEA suppresses insulin-like growth factor-1 (IGF-1), a hormone that normally helps ovarian function [30]. Both decreased leptin (an adipose signal of energy stores) and reduced IGF-1 levels from energy deficit help explain why the hypothalamus downregulates reproduction. Leptin levels fall quickly with LEA, which signals to the hypothalamus that energy stores are low [30,31]. Low leptin levels contribute to reduced GnRH/LH and oestradiol levels in women, causing menstrual disturbances if prolonged.

Other hormones change in predictable ways. Thyroid function slows: T3 (active thyroid hormone) drops since the body does not want to burn unnecessary energy [30]. In fact, Loucks and Heath found that a low-T3 syndrome develops once EA falls below a critical threshold [25]. Cortisol often rises as part of the stress response to energy shortage [26,31]. Levels of growth hormone (GH) and its pulsatility may actually increase, but this reflects a compensatory attempt to mobilise fat and maintain blood sugar [26,30]. By contrast, insulin levels usually fall when LEA is present, reflecting lower glucose intake and higher fat metabolism [31,94].

Sex hormones also respond differently. In females, oestrogen (oestradiol) levels decline when LH pulses diminish, contributing to amenorrhea over time [26,30]. In males, short bouts of low EA may not immediately suppress testosterone, but severe or chronic LEA will eventually lead to lower testosterone levels and disturbed sperm parameters as the pituitary–gonadal axis senses an energy crisis. One study in male athletes found no acute drop in testosterone or T3 levels after 4 days of severe LEA, but leptin and insulin levels did fall markedly [92]. This suggests the male reproductive system is somewhat more resilient in the short term, but chronic LEA can still impair testosterone and fertility.

In summary, low energy availability causes an “energy-conservation” endocrine profile: lower leptin, insulin, IGF-1, thyroid hormone, and oestrogen/testosterone levels alongside higher cortisol, GH, and ghrelin (the “hunger hormone”) levels [26,30,31]. All of these shifts signal the body to slow growth, reproduction, and metabolism when calories are scarce.

### 3.2. Metabolic Adaptations to Low Energy Availability

Energy deficiency also forces metabolic changes to prioritise essential functions. One key adaptation is a reduction in the resting metabolic rate (RMR). The body lowers energy expenditure at rest, so fewer calories are needed for the same basic functions [30,31]. This slowing of metabolism is partly due to lower thyroid hormone levels and partly due to the loss of metabolically active tissue. In other words, the body intentionally becomes more fuel-efficient when energy intake is low.

Substrate utilisation shifts as well. With less carbohydrate intake or availability, the body increases fat oxidation and ketone production to feed the brain and muscles. Studies report higher fatty acid and ketone levels in the blood during low EA as insulin is suppressed and lipolysis increases [31]. Glucose levels may drop, and the body “spares” glucose for the brain by burning more fat. Over time, muscles also become insulin-resistant in a way, reducing glucose uptake and signalling tissues to switch fuel sources. These changes help maintain blood sugar but also reflect a shift to a catabolic (breakdown) state.

At the cellular level, mitochondrial efficiency changes. There may be less mitochondrial uncoupling (less heat production), and energy in cells is conserved. In sum, the whole-body metabolic rate falls, and substrate usage adapts so that energy intake matches the new lower expenditure. While this helps survival, it can impair performance. Athletes with LEA often feel lethargic, lose endurance and strength, and have higher perceived exertion during exercise, even if body weight falls [30,31].

Another metabolic change is in hunger hormones: ghrelin (which stimulates appetite) levels tend to rise, and leptin (which suppresses appetite) levels fall, which is consistent with the body’s attempt to encourage eating [31]. These appetite signals are part of the feedback that the body is in energy deficit. Overall, the hormonal changes and metabolic adaptations create a situation where energy is conserved and redirected away from growth and reproductive functions toward survival.

### 3.3. Effects on Body Composition and Performance

As a result of hormonal and metabolic adaptations, body composition shifts under LEA. Lean mass (muscle) is often sacrificed. The hormonal milieu—with lower insulin, IGF-1, and thyroid hormone levels alongside higher cortisol levels—is catabolic, meaning it promotes muscle protein breakdown. Indeed, studies show that short-term severe LEA (e.g., 14 days at ~22 kcal/kg FFM) leads to measurable losses in fat-free mass despite adequate protein and resistance exercise [30]. Muscle protein synthesis rates drop significantly when calories are inadequate, even if protein intake is high [30]. Thus, athletes may lose muscle strength and power during sustained energy deficit.

Fat mass often decreases as well, which is usually the goal of weight loss. However, after the deficit ends, the body may rapidly regain fat to restore its energy reserves. Some evidence suggests an increased propensity to store fat once normal eating resumes due to metabolic “overshoot.” Bone density and tendon health can also suffer since collagen synthesis is hormonally regulated and requires calories; chronic LEA raises bone resorption markers [30].

In practical terms, changes in body composition influence performance. A lighter body weight might improve relative power (e.g., runners and cyclists), but the loss of muscle strength, endurance, and coordination often outweighs this in the short term. Moreover, low energy availability commonly leads to fatigue, mood disturbances, and injuries (e.g., stress fractures and tendon injuries) due to weakened tissue and immune changes. Performance metrics in studies reflect this: athletes under LEA show reduced power output, slower times, and impaired recovery [30,31]. Even in men, where hormones may seem stable in the short term, decreased energy undermines endurance and sprint measures [93]. Thus, both hormonal and metabolic responses to LEA negatively impact body composition and function in the real world.

In summary, exercise-induced low energy availability leads to a coordinated hormonal response and metabolic slowdown. Levels of key hormones like leptin, insulin, thyroid hormones, IGF-1, and sex steroids fall, while those of cortisol and ghrelin rise [26,30,31]. These changes reduce the RMR and shift fuel use toward preserving brain and essential organs. Over time, muscle and bone suffer, and reproductive function is suppressed. Clinical recognition of REDs is important because even short-term energy deficits can have significant consequences for health and performance [2,31].

## 4. Exercise-Induced Relative Energy Deficiency: Body Composition, Structural Integrity, and the Role of Nutrition

Relative energy deficiency in sport (REDs) has become an essential concept in sports medicine, describing the spectrum of physiological impairments that occur when an athlete’s energy intake does not match the demands of both exercise and basal metabolism.

What makes REDs particularly insidious is its gradual onset. Athletes may continue to train and compete while experiencing subtle hormonal disturbances, compromised bone health, and recurrent injuries. The syndrome only becomes clinically obvious when overt symptoms such as amenorrhea, stress fractures, or performance plateaus emerge. The present review examines how REDs alters body composition and structural integrity and discusses how nutrition can prevent and correct these changes, with practical recommendations for athletes, coaches, and sports nutrition professionals [17].

### 4.1. REDs and Body Composition

As established in Section 2.1 and Section 3.1, low EA leads the body to downregulate processes that are not essential to immediate survival, including reproduction and growth pathways, with luteinising hormone pulsatility suppressed below approximately 30 kcal·kg FFM^−1^·day^−1^ [25,26].

These adaptations extend beyond the reproductive system. Recreational runners with chronically low EA have been shown to exhibit high rates of luteal phase deficiency and anovulation [75], illustrating that energy deficiency is not confined to elite sport but also affects active individuals. At the same time, reduced levels of triiodothyronine (T3) and leptin decrease the resting metabolic rate, conserving energy by suppressing thermogenesis.

**Figure 5:**
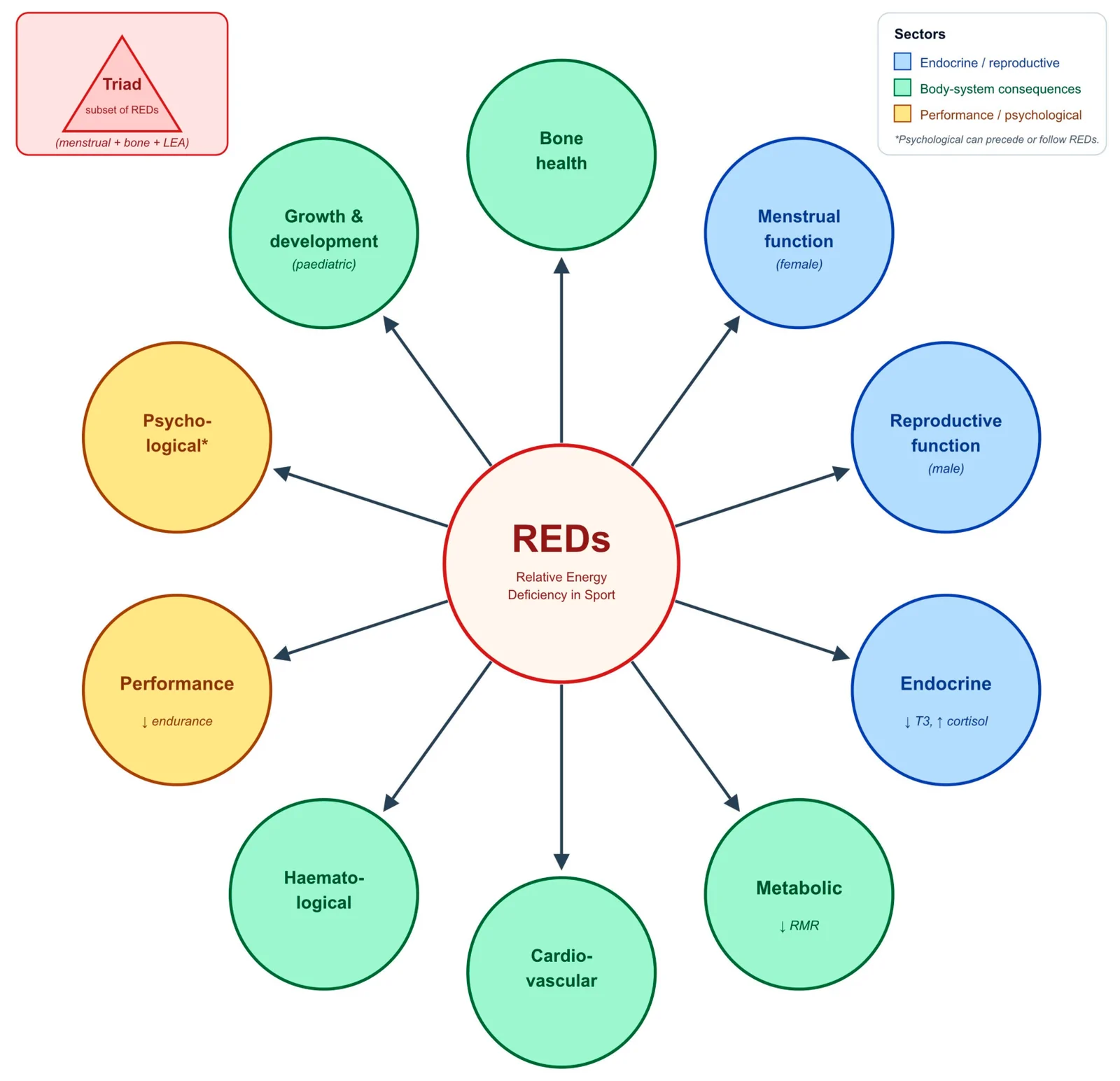
Health consequences of relative energy deficiency in sport (REDs) showing an expanded concept of the female athlete triad to acknowledge a wider range of outcomes and the application to male athletes (psychological consequences can either precede REDs or be the result of REDs). Arrows radiating from the central REDs node denote a causal relationship with each affected body system or domain; downward (↓) and upward (↑) arrows within circles denote a decrease or increase in the indicated parameter, respectively. Figure created by the authors; the concept is based on ref. [95].

For body composition, these changes often lead to a paradoxical outcome. Athletes may fail to build lean muscle mass despite intensive training while retaining central adiposity. The IOC’s 2023 consensus statement emphasises that inadequate carbohydrate availability can independently blunt muscle protein synthesis, impair training adaptations, and exacerbate neuroendocrine stress, even if total caloric intake appears sufficient [2]. This underscores the importance of both energy sufficiency and nutrient timing in maintaining body composition and performance (Figure 5).

### 4.2. Bone Health

The hormonal mechanism by which low EA shifts bone metabolism toward resorption is set out in Section 2.2 and is not repeated here; its net effect is reduced bone mineral density, altered microarchitecture, and increased fracture risk [96].

Clinical studies highlight the seriousness of these effects. Female high school runners of all ages with menstrual dysfunction (14–18 years old) experienced significantly higher rates of stress fractures compared with eumenorrheic athletes, providing strong evidence for the link between REDs and compromised bone health [94]. In military training environments, calcium and vitamin D supplementation has been shown to reduce stress fracture incidence by approximately 20% [97], although this cannot fully compensate for inadequate EA. These findings emphasise that nutritional sufficiency is necessary but not sufficient: restoring energy balance is the primary determinant of skeletal integrity.

### 4.3. Connective Tissue and Muscle

The consequences of REDs extend to connective tissue and muscle. Inadequate energy and protein intake impair collagen synthesis and delay muscle repair, predisposing athletes to recurrent soft-tissue injuries and prolonged recovery. The IOC’s most recent consensus highlights that chronic low EA often leads to persistent soreness, stagnating performance, and increased risk of overuse injuries [2]. This cycle can frustrate athletes, who may respond by increasing training loads, thereby worsening the underlying energy deficit.

## 5. Nutritional Strategies for Prevention and Recovery

### 5.1. Restoring Energy Availability

The cornerstone of REDs management is restoring EA. The IOC emphasises that nutritional rehabilitation is the first-line treatment, while pharmacological interventions should be considered only in specific cases [2]. Evidence from the REFUEL trial demonstrated that increasing energy intake by approximately 330 kcal/day over twelve months doubled the likelihood of menstrual recovery in women with exercise-induced amenorrhea [98]. This suggests that even relatively modest increases in energy intake can restore endocrine function and improve health outcomes.

In practice, strategies for restoring EA include adding energy-dense snacks before and after training, increasing portion sizes, and reducing excessive training loads when necessary. Importantly, interventions should be sustainable and tailored to the athlete’s lifestyle, dietary preferences, and training schedule.

### 5.2. Carbohydrate Availability and Timing

Carbohydrates are a key nutrient for athletes, not only for fuelling exercise but also for protecting endocrine and immune function. Research in elite marathoners demonstrated that carbohydrate periodisation—adjusting intake to match session intensity and duration—helps optimise performance while preventing chronic low carbohydrate availability. Burke et al. [99] noted that while occasional “train low” sessions may stimulate mitochondrial adaptations, chronically low carbohydrate availability impairs immune function, blunts training adaptations, and increases REDs risk.

Practical carbohydrate strategies include consuming 1–4 g/kg body mass in the 1–4 h before high-intensity exercise, ingesting 30–60 g per hour during prolonged training, and replenishing glycogen with 1.0–1.2 g/kg within the first two hours post-exercise. These practices help ensure that athletes do not repeatedly train in a depleted state, thereby reducing REDs risk [100].

### 5.3. Protein Intake and Distribution

Protein is essential for muscle and connective tissue health. A meta-analysis demonstrated that daily intakes of around 1.6 g/kg body mass optimise muscle protein synthesis [101], while higher intakes of up to 2.2 g/kg may help mitigate muscle loss during periods of energy deficit. Distribution across the day matters: consuming 0.3–0.4 g/kg protein every 3–4 h is more effective than skewed intake [45].

Practical strategies for athletes include incorporating protein into every meal and snack. Examples include Greek yogurt at breakfast, lean poultry or legumes at lunch, a protein shake post-training, and fish or eggs at dinner. Athletes recovering from injuries may benefit from slightly higher protein intake within the recommended range to support tissue remodelling. Additionally, collagen-rich foods or gelatine combined with vitamin C may aid tendon and ligament repair.

### 5.4. Micronutrients

Micronutrients complement macronutrient strategies. Calcium and vitamin D are critical for bone health, and supplementation has been shown to reduce stress fractures in military recruits. Iron deficiency is another frequent issue, particularly in endurance athletes and women. Sim et al. [102] reviewed evidence showing that correcting iron deficiency improves maximal oxygen uptake and endurance performance. Screening for ferritin and haemoglobin should therefore be routine in high-risk populations.

While supplementation can be useful, athletes should prioritise nutrient-rich diets: dairy products, leafy greens, oily fish, and lean red meat are excellent sources of calcium, vitamin D, and iron. Supplementation should be targeted and supervised to avoid excess intake or interactions.

### 5.5. Screening and Practical Recommendations

Early detection of REDs is critical for prevention. The Low Energy Availability in Females Questionnaire (LEAF-Q) has been validated for identifying women at risk of REDs-related dysfunctions. To address both sexes and broader contexts, the IOC recently introduced the REDs Clinical Assessment Tool (CAT2), which stratifies athletes into low-, moderate-, and high-risk categories, guiding return-to-play decisions [2].

Practical approaches for prevention include increasing daily energy intake by 300–600 kcal when early symptoms appear, consuming carbohydrates before and after training, avoiding frequent fasted high-intensity sessions, and ensuring adequate protein intake distributed evenly across the day [88,96]. Athletes should also monitor micronutrient intake and consider supplementation when deficiencies are detected.

The tools available for screening and monitoring differ considerably in how well they are validated, and we distinguish strongly supported measures from consensus-based guidance and from proposals that remain poorly validated. Table 4 sets out a practical screening panel on that basis; hydration markers, in particular, are included because hypohydration is plausibly and, on emerging evidence, associatively linked to REDs risk, not because their use in REDs staging has been validated.

Education at the organisation level is equally important. Nutrition education combined with individualised counselling has been shown to reduce REDs symptoms and improve fuelling behaviours among endurance athletes. Coaches play a central role in shaping team culture and should emphasise fuelling, recovery, and long-term health rather than body composition alone.

Recovery from REDs varies considerably. Menstrual function may return within 6–12 months of restoring EA, but bone health improvements often take years [96]. Monitoring should include both clinical symptoms (e.g., menstrual function, libido, and fatigue) and laboratory values (e.g., ferritin, vitamin D, and sex hormones). Athletes should resume full training and competition only when health markers improve, not solely based on performance metrics.

REDs is a complex condition that threatens both athlete health and performance. Its effects on body composition—impaired lean mass development and paradoxical fat distribution—and structural integrity—reduced bone density, altered bone microarchitecture, and impaired tissue repair—underscore its systemic impact. At the same time, nutrition offers the most powerful tool for both prevention and recovery. Restoring energy availability, periodising carbohydrate intake, optimising protein distribution, and addressing micronutrient deficiencies can reverse many of the impairments caused by REDs.

Screening tools like the LEAF-Q and the REDs CAT2 support early detection, while educational and organisational strategies help to build supportive environments. Ultimately, preventing REDs requires collaboration between athletes, coaches, nutritionists, and medical professionals, alongside cultural change in sport to prioritise fuelling and long-term health as much as performance.

### 5.6. Red Flags and Intervention Strategies

Table 5 summarises the principal clinical red flags associated with REDs, alongside evidence-based intervention strategies and recommended monitoring tools to guide early detection and multidisciplinary management.

### 5.7. Multidisciplinary Management Model

REDs demand integrated care:

- Sports dietitian: Custom EA/macronutrient plans (e.g., carb periodisation and protein distribution) and micronutrient screening.
- Physician/endocrinologist: Hormone/bone labs (e.g., ferritin, vitamin D, and sex hormones) and pharmacology if needed.
- Psychologist: ED/DE screening/treatment and behavioural support.
- Coach/physiotherapist: Load management, recovery monitoring, and performance tracking.
- Athlete input: Lifestyle/preference alignment.
- Periodic reassessment with the CAT2, supported by the LEAF-Q or its male equivalent for screening, tracking recovery over 6–12 months (e.g., menstrual return and gains in bone mass); these tools are intended for screening and clinical staging rather than frequent administration, and the interval should be set clinically rather than fixed weekly [96].

## 6. Discussion

The present review synthesised how exercise-induced low energy availability (LEA) alters body composition and physiological function, particularly in athletes exposed to high training loads. The retrieved evidence was broadly consistent in indicating that insufficient energy intake impairs multiple physiological systems, although the magnitude and temporal course of these effects differ across studies and disciplines. The following discussion evaluates these findings in light of the review objective, identifying convergences, contradictions, and methodological strengths and limitations within the literature.

Because the evidence base is markedly uneven across the four compartments, we grade it explicitly here rather than presenting REDs as producing uniform or coordinated change. **Bone: strong.** Dose–response experimental data in exercising women [42], consistent cohort findings across disciplines, and consensus endorsement [2] converge on the same direction of effect. **Skeletal muscle and endocrine function: strong to moderate.** Controlled feeding and stable-isotope studies show concordant reductions in myofibrillar protein synthesis and in anabolic hormone concentrations [28,46], although sample sizes are small and the participants are predominantly female. **Total and visceral adiposity: indirect.** No study has quantified adipose depots in athletes stratified by measured energy availability; the available data were derived from overweight and obese non-athletic cohorts and from animal models [62,63,64,65,66,67]. **Hydration: emerging and associative.** Recent survey and case evidence links inadequate hydration to REDs risk [79,80], but no study has manipulated energy availability and hydration status independently. Conclusions in the latter two domains are therefore advanced as hypotheses requiring targeted testing and should not be read as established findings.

### 6.1. Consistency and Divergence Across Studies

Across most investigations, the detrimental influence of LEA on bone density, muscle mass, and hormonal balance is well established. Numerous studies demonstrate that inadequate energy intake reduces bone formation and enhances bone resorption, predisposing athletes to stress fractures and reduced bone mineral density [26,42]. Similarly, suppressed anabolic hormones, such as IGF-1 and testosterone, coincide with slower muscle protein synthesis and loss of lean tissue [28,45]. These findings broadly support the hypothesis that chronic energy deficit disrupts structural integrity and metabolic function.

However, the pace and extent of these changes are not uniform. Some laboratory studies reported measurable physiological disruption within just a few days of caloric restriction, whereas field-based or longitudinal data suggest that adverse outcomes develop gradually over weeks or months [30]. This variation likely reflects differences in study design, sample size, and the degree of energy restriction imposed. Gender also contributes to variation: female athletes tend to exhibit earlier and more pronounced hormonal suppression than males, whose reproductive hormones are typically affected only after prolonged LEA [93]. These discrepancies highlight the complex interplay between sex, training intensity, and nutritional behaviour in shaping physiological responses.

Findings regarding body fat and hydration are more fragmented. While overall fat mass typically declines under sustained LEA, evidence about changes in visceral fat remains inconsistent. Some authors describe substantial hormonal disruption associated with excessive fat loss [17], whereas others report minimal changes in visceral distribution when deficits are moderate [2]. Evidence linking LEA or REDs to fluid balance remains sparse but is no longer absent: inadequate hydration has been identified as a significant predictor of REDs risk among physically active individuals, most clearly in men [79], and case material from weight-making athletes shows that severe energy restriction and deliberate dehydration are frequently incurred together and can converge on hypernatraemia and acute kidney injury [80]. The bulk of the hydration literature nevertheless still concerns dehydration and performance in general athletic populations rather than energy-deficient states [19], and no study has manipulated the two exposures independently, so the direction of causality—whether hypohydration deepens an energy deficit or merely travels with it—remains undetermined. These uncertainties emphasise the need for more integrated studies that evaluate all components of body composition simultaneously.

### 6.2. Critical Review of Methodological Approaches

Research on REDs and LEA benefits from a broad interdisciplinary foundation that draws on endocrinology, nutrition, exercise physiology, and morphology. The transition from the female athlete triad model to the REDs framework reflects a major conceptual advance, acknowledging that low energy availability affects multiple systems and both sexes. Modern studies often use advanced technologies—such as doubly labelled water, isotope tracers, and DXA scanning—to provide detailed insight into energy flux and tissue composition.

Despite this progress, methodological challenges persist. Energy availability is notoriously difficult to measure accurately, as both dietary intake and energy expenditure rely heavily on self-reporting and indirect calculations [32]. Many studies also feature small cohorts, short intervention periods, or narrow participant groups, which limits their generalisability. Furthermore, most experimental work focuses on females due to historical interest in menstrual dysfunction, leaving significant knowledge gaps regarding male physiology. Another recurring limitation is the inconsistent definition of “low” energy availability—some research expresses it as a fixed caloric value, while other studies adjust for fat-free mass, complicating comparisons across studies.

### 6.3. Advantages and Shortcomings of Existing Evidence

One of the main advantages of current research is its recognition of REDs as a systemic syndrome rather than an isolated reproductive or skeletal issue. This shift has prompted broader screening and prevention initiatives and improved the understanding of how energy deficiency interacts with stress hormones, metabolism, and immune function. Furthermore, controlled trials such as those by De Souza et al. [98] provide concrete evidence that even modest increases in caloric intake can restore endocrine health, reinforcing the practical importance of adequate fuelling strategies.

Nonetheless, several weaknesses remain. A large portion of the available data is correlational rather than causal, meaning it is often unclear whether observed changes result directly from LEA or from associated factors such as overtraining, psychological stress, or nutrient deficiencies. Additionally, the predominance of short-term studies does not capture long-term adaptations or the cumulative health burden of chronic low energy intake. Disordered eating behaviours, a major driver of LEA in aesthetic and endurance sports, are frequently underreported and thus underestimated. Addressing these gaps will require larger, multi-centre studies that combine physiological, behavioural, and psychological assessment.

### 6.4. Integration with the Review Objectives and Question

The collective evidence addresses the central question and objectives of this review: namely, the retrieved literature is consistent with exercise-induced LEA being associated with measurable reductions in muscle mass and bone mineral density and with hormonal disturbance, alongside less certain effects on hydration and metabolic regulation. The data further confirm that the severity and manifestation of these effects differ by sport type. Endurance and aesthetic athletes, who often sustain chronic caloric deficits, are most vulnerable to long-term consequences such as bone stress injuries and hormonal dysfunctions. Strength athletes typically experience more transient episodes of LEA—such as during weight-cutting phases—but may still suffer acute declines in power output and recovery capacity. These observations align with the notion that energy availability below roughly 30 kcal/kg FFM/day consistently predicts physiological compromise, while values above 45 kcal/kg FFM/day appear protective.

Additionally, monitoring body composition and physical load is fundamental for identifying athletes at risk of REDs. There are various assessments for those factors using a combination of objective and subjective methods. Objective measurements can include power output, heart rate monitoring, and GPS tracking of factors such as speed, distance, and elevation, which together quantify the external workload. Wearable technology integrating accelerometery and heart rate provides reliable estimates of exercise energy expenditure in field settings [103]. Subjective measures include assessments like session rate of perceived exertion (sRPE). They are used frequently when there is a necessity to capture the athlete’s perceived internal load and complement objective data [103]. To assess the physiological impact of energy deficiency, body composition monitoring is essential. The gold standard for the evaluation of fat/lean mass and bone mineral density is dual energy x-ray absorptiometry (DXA), which is used in REDs research [96]. Bioelectrical impedance analysis (BIA) and air-displacement plethysmography are offered as alternative assessment types, even though these methods are not as precise as DXA but are used when performing DXA is not possible [104]. Regular tracking allows early detection of maladaptive responses to energy deficiency and supports individual nutrition and recovery plans.

## 7. Conclusions

Chronic LEA is the proximate driver of REDs, but the strength of the supporting evidence differs markedly between compartments and the conclusions below are calibrated accordingly. It is well established that sustained LEA reduces bone mineral density and skeletal muscle mass and disturbs endocrine function in a sport-dependent manner, with endurance and aesthetic athletes most severely affected and weight-category athletes exposed intermittently through acute weight-making practices. By contrast, effects on total and visceral adiposity remain indirect and have never been quantified in athletes stratified by measured energy availability, and the relationship between LEA and hydration status is emerging and associative rather than established. Conclusions in these two domains are advanced as hypotheses requiring targeted testing, not as findings.

From a translational standpoint, EA at or above 45 kcal·kg FFM^−1^·day^−1^ is a commonly cited reference point rather than a universal minimum, given the substantial measurement error in estimating EA and the wide individual variation in the threshold at which function is disturbed. Alongside the LEAF-Q or male-equivalent screening, confirmatory CAT2 staging, and domain-targeted biomarker follow-up (e.g., RMR, T3, reproductive hormones, IGF-1, bone-turnover markers, and DXA), it offers a practical framework for protecting athlete health.

Priority research gaps concern male and adolescent athletes, within-day versus 24 h EA dynamics, direct quantification of visceral adipose tissue in athletes stratified by measured EA, controlled trials dissociating hypohydration from energy deficiency, and dose–response trials quantifying the duration of restored EA required for biological recovery.

## Figures and Tables

**Table 1:** Summary of article screening process.

Screening Stage	Action	Remaining
Records identified	Initial database search	142
Duplicates removed	Cross-database deduplication	97
Title/abstract screening	Relevance to LEA/REDs and body composition	82
Full-text review	Inclusion/exclusion criteria applied	63
Updated search (March 2025–June 2026)	Additional records identified, screened and included	85

**Table 2:** Summary of principal included studies (selected evidence underpinning the discussion on LEA, REDs and body composition).

Study (Author, Year)	Design	Population	Key Finding/Relevance
Mountjoy et al. [2]	IOC consensus statement	Elite and recreational athletes (both sexes)	Updated IOC REDs framework; introduced CAT2 clinical assessment tool for staging severity.
Gallant et al. [3]	Narrative review	Athletes with LEA/REDs	Synthesised current understanding of REDs mechanisms across systems.
Puscheck, Kennel & Saenz [4]	Cross-sectional study	Collegiate athletes (*n* = multi-site)	Quantified concurrent prevalence of disordered-eating risk and LEA risk.
Stellingwerff & Hamilton [9]	Narrative review	Endurance and aesthetic-sport athletes	Delineated overlap between Overtraining Syndrome and REDs; identified shared endocrine pathways.
Angelidi et al. [10]	Narrative review	Athletes with low energy availability	Updated pathophysiology of endocrine manifestations; treatment-oriented framework.
Nattiv et al. [13]	Consensus statement	Male athletes	Defined the male athlete triad (LEA, suppressed reproductive function, and impaired bone health).
Heymsfield et al. [16]	Critical methodological review	Body composition assessment	Clarified distinction between fat-free mass and lean body mass; DXA limitations.
Heikura, Stellingwerff & Areta [27]	Narrative review	Female athletes	Critical appraisal of laboratory vs. field measurement of energy availability.
Oxfeldt et al. [28]	Randomised crossover trial	Eight trained women	Five days of LEA (~25 kcal·kg FFM^−1^·day^−1^) impaired myofibrillar protein synthesis.
Jeppesen et al. [29]	Experimental trial (crossover)	Endurance-trained males	Short-term severe LEA increased oxidative stress; antioxidant capacity decreased.
Jeppesen et al. [30]	Molecular/endocrine study	Trained athletes (mixed sex)	Characterised short-term severe LEA signatures at molecular, endocrine, and performance levels.
Nolte et al. [31]	Controlled intervention	Male endurance athletes	Three-day LEA altered substrate metabolism and produced early performance decrements.

**Table 3:** Sport-specific risk of relative energy deficiency in sport (REDs).

Sport Type	REDs Risk Level	Key Risk Factors	Common Consequences	Strength of Evidence
Endurance (distance running, cycling, triathlon, cross-country skiing)	Very high	High exercise energy expenditure; prolonged training; unintentional under-fuelling	Menstrual dysfunction, bone stress injuries, suppressed RMR, impaired aerobic performance	Strong: multiple cohorts, dose–response experimental data and consensus endorsement
Ultra-endurance	Very high	Extreme and only partially replaceable energy cost; sociocultural emphasis on leanness	LEA, disordered eating, psychological stress	Moderate: few primary cohorts; largely review and qualitative evidence
Aesthetic and weight-category (gymnastics, figure skating, dance, bodybuilding, combat sports)	High	Judged or regulated body mass; body-image pressure; intentional caloric and fluid restriction	Disordered eating, endocrine disturbance, low BMD; dehydration and acute kidney injury with extreme weight-making	Moderate–strong for LEA and disordered-eating prevalence; limited for direct body-composition endpoints
Speed and power	Moderate–high	Intense training with episodic energy restriction; deficit often masked by favourable power-to-mass ratio	Bone stress injuries, reduced BMD, suppressed anabolic signalling and impaired recovery	Moderate: single national screening cohort; mechanistic support from tracer studies
Team sports (volleyball, netball, football)	Moderate	High training volume; low awareness of REDs; little routine screening	LEA markers, moderate injury risk, delayed recognition	Limited: few cohorts, single-sport samples, no longitudinal data

**Table 4:** Practical screening and monitoring panel for REDs, graded by strength of supporting evidence.

Domain	Tool or Marker	When to Use	Strength of Evidence
Energy availability	EA calculation (EI − EEE per kg FFM); food and training logs	Baseline; whenever symptoms appear	Consensus-based; measurement error in EI and EEE is substantial
First-line screening	LEAF-Q (females); LEAM-Q (males)	All athletes in at-risk disciplines, seasonally	Validated in females; the male equivalent is less well validated
Severity staging	IOC REDs Clinical Assessment Tool (CAT2)	After a positive screen; to guide return to play	Consensus-based [2]
Body composition and bone	DXA (fat mass, lean mass, BMD); BIA or ADP where DXA is unavailable	Baseline and 6–12-monthly	Strongly supported; DXA is the reference standard [16,96]
Reproductive and endocrine	Menstrual history; oestradiol, testosterone, LH, T3, IGF-1, cortisol	On clinical suspicion of REDs	Strongly supported in females; more limited normative data in males
Metabolic	Measured RMR and RMR ratio	Where metabolic suppression is suspected	Moderate; diagnostic thresholds are not standardised
Haematological and micronutrient	Ferritin, haemoglobin, vitamin D	Routine in endurance athletes and in females	Strongly supported [102]
Hydration	Urine specific gravity or osmolality; body-mass change across sessions	Weight-category and aesthetic athletes, around weigh-in	Poorly validated in the REDs context; proposed on emerging associative evidence [79,80], not established

**Table 5:** Red flags and intervention strategies.

Red Flags	Intervention Strategies	Monitoring Tools
Menstrual dysfunction, low libido, fatigue	Increase EA by 300–600 kcal/day (330 kcal as in REFUEL); add energy dense snacks pre/post-training.	LEAF-Q, CAT2, sex hormones
Frequent injuries/stress fractures	Boost calcium/vitamin D (diet/supplements); prioritise bone health	DXA scan, vitamin D/ferritin
Performance plateau, GI issues	Periodise carbs (1–4 g/kg pre-exercise, 30–60 g/h during, 1.0–1.2 g/kg post)	Training logs, glycogen markers
Elevated RMR suppression	Reduce training loads; restore EA gradually	RMR testing, EA calculations
Immune suppression, frequent illness	Avoid chronic “train-low”; ensure carb fuelling for endocrine/immune protection	Immune markers, infection logs
Muscle loss, poor recovery	Protein 1.6–2.2 g/kg/day, distributed 0.3–0.4 g/kg/meal; collagen + vitamin C for tendons	FFM via DXA, injury history
Iron deficiency (esp. endurance/females)	Screen ferritin/haemoglobin; nutrient-rich diet + targeted iron supplements	Ferritin, haemoglobin levels
Chronic Low EA symptoms (e.g., skewed intake)	Tailored plans: portion increases, sustainable snacks; education	EA questionnaire, weight trends

## Data Availability

No new data were created or analyzed in this study.

## Abbreviations

The following abbreviations are used in this manuscript:

BC	Body composition
BMD	Bone mineral density
CAT	Clinical Assessment Tool
DE	Disordered eating
DEI	Dietary energy intake
EA	Energy availability
EB	Energy balance
ED	Eating disorder
EEE	Exercise energy expenditure
EI	Energy intake
FFM	Fat free mass
FSR	Fractional synthetic rate
GH	Growth hormone
IOC	International Olympic Committee
LEA	Low energy availability
LEAF-Q	Low energy availability in females questionnaire
LH	Luteinizing hormone
OEA	Optimal energy availability
REDs	Relative energy deficiency in sport
RMR	Resting metabolic rate
TEE	Total energy expenditure
DXA	Dual-energy X-ray absorptiometry
BIA	Bioelectrical impedance analysis
ADP	Air-displacement plethysmography
MET	Metabolic equivalent of task
LEAM-Q	Low energy availability in males questionnaire
